# Impaired Interleukin-1β and c-Fos Expression in the Hippocampus Is Associated with a Spatial Memory Deficit in P2X_7_ Receptor-Deficient Mice

**DOI:** 10.1371/journal.pone.0006006

**Published:** 2009-06-23

**Authors:** Virginie F. Labrousse, Laurence Costes, Agnès Aubert, Muriel Darnaudéry, Guillaume Ferreira, Thierry Amédée, Sophie Layé

**Affiliations:** 1 Psychoneuroimmunologie, Nutrition et Génétique (PsyNuGen), INRA UMR 1286, CNRS UMR 5226, Université de Bordeaux, Bordeaux, France; 2 NEUROSTRESS EA 4347, “Université Lille Nord de France”, Villeneuve d'Ascq, France; 3 Laboratoire Comportement, Neurobiologie et Adaptation, INRA UMR 85, CNRS UMR 6175, Université de Tours, Nouzilly, France; James Cook University, Australia

## Abstract

Recent evidence suggests that interleukin-1β (IL-1β), which was originally identified as a proinflammatory cytokine, is also required in the brain for memory processes. We have previously shown that IL-1β synthesis in the hippocampus is dependent on P2X_7_ receptor (P2X_7_R), which is an ionotropic receptor of ATP. To substantiate the role of P2X_7_R in both brain IL-1β expression and memory processes, we examined the induction of IL-1β mRNA expression in the hippocampus of wild-type (WT) and homozygous P2X_7_ receptor knockout mice (P2X_7_R^−/−^) following a spatial memory task. The spatial recognition task induced both IL-1β mRNA expression and c-Fos protein activation in the hippocampus of WT but not of P2X_7_R^−/−^ mice. Remarkably, P2X_7_R^−/−^ mice displayed spatial memory impairment in a hippocampal-dependant task, while their performances in an object recognition task were unaltered. Taken together, our results show that P2X_7_R plays a critical role in spatial memory processes and the associated hippocampal IL-1β mRNA synthesis and c-Fos activation.

## Introduction

The interleukin-1 (IL-1) family is composed of three closely-related proteins that are products of different genes: two agonists, IL-1α and IL-1β, and a naturally-occurring IL-1 receptor antagonist (IL-1ra) [1]. All the members of this family are synthesized and/or released in the brain by both glial cells and neurons [2]. Although most of the evidence gathered so far indicates that pathophysiological levels of IL-1 detrimentally affects neural plasticity and memory processes [3], recent evidence suggests that this cytokine is required for the physiological regulation of memory processes [4]–[7]. Blocking IL-1 receptors with IL-1ra impairs the maintenance of long-term potentiation (LTP) and hippocampal-dependent memory performance as measured in water maze and passive avoidance paradigms [7]. IL-1 receptor knockout mice exhibit a marked inability to express LTP in the hippocampus [4]. IL-1β, rather than IL-1α, could be involved in memory processes, since IL-1β administration improves avoidance memory and contextual fear conditioning [7]. This is further reinforced by the observation that hippocampal IL-1β mRNA expression is increased substantially after contextual fear conditioning and after LTP both *in vitro* and *in vivo* [5], [6].

Extracellular ATP has been reported to trigger the release of IL-1β by binding to type 2 purinergic receptors (P2 receptors) [8]. These receptors are subdivided into two subgroups: P2Y metabotropic receptors (P2YR), which are G protein-coupled receptors, and P2X ionotropic receptors (P2XR), which are ligand-gated ion channels [9]. Interestingly, microglial cells, which are the main cell source of IL-1β in the brain [2], express P2X_7_R [10], [11]. ATP binds to P2X_7_R to induce the activation of caspase-1, the IL-1β processing enzyme, also releasing mature IL-1β in the process [8]. We and others have demonstrated *in vitro* that ATP triggers the maturation and release of IL-1β in response to the bacterial endotoxin lipopolysaccharide (LPS) in microglia [12], [13]. *In vivo*, LPS-induced IL-1β mRNA expression is significantly lower in the brain of P2X_7_R^−/−^ mice compared with WT mice [12].

Although the last decade has seen a wealth of knowledge on the effects of IL-1β in the hippocampus, much less is known about the mechanism of IL-1β release under physiological conditions, especially during hippocampal-dependent memory processes. We have recently identified P2X_7_R as a key component in LPS-mediated mature IL-1β release by microglia [12], and it has been shown that IL-1β synthesis is induced in the hippocampus by memory processes [5]. Hence, the aim of the present study was to investigate the role of P2X_7_R in memory processes and hippocampal IL-1β production. For this purpose, we examined the memory performances of P2X_7_R^−/−^ mice in two spontaneous recognition paradigms (Y maze and object recognition). Our results show that after 30 minutes of retention, spatial memory was impaired in P2X_7_R^−/−^ mice, while their performances were unaltered in an object recognition memory task. Moreover, the induction of IL-1β synthesis and c-Fos expression in the hippocampus in response to the spatial task was abolished in P2X_7_R^−/−^ mice, suggesting that hippocampal IL-1β production may be involved in spatial memory performance.

## Materials and Methods

### Animals

Animal experiments were carried out according to the INRA Quality Reference System (1), and to relevant French (Directive 87–148, Ministère de l'Agriculture et de la Pêche) and international (Directive 86–609, November 24^th^ 1986, European Community) legislation. Every effort was made to minimize suffering and the number of animals used. Experiments were performed on 3-month-old C57BL/6 (WT, n = 33) and homozygous P2X_7_R^−/−^ (n = 33) male mice weighing 25–30 g. The P2X_7_R^−/−^ mice were raised on a C57Bl/6 background and were kindly provided by Dr. Gabel (Pfizer, Groton, USA) [14]. Lack of the P2X_7_ receptor was confirmed in the P2X_7_R^−/−^ mice using PCR (not shown), as previously described [15]. P2X_7_R^−/−^ and C57BL/6 mice were raised in the laboratory [12] and kept in groups of three in transparent polycarbonate cages (26.6×21.4×14.3 cm). They were maintained under standard colony conditions on corn cob litter in a temperature- (23±1°C) and humidity- (40%) controlled animal room under a 12 h light/dark cycle (7:00–19:00), with *ad libitum* access to food and water.

### Behavioral measurements

Behavioral testing took place in the morning (between 8:00 AM and 11:00 AM). Mice were first handled for 5 min every day two weeks before launching the experiments. All the experiments were performed in a room adjacent to the *vivarium* under 78 *lux* of illumination.

#### Spatial recognition

The spontaneous spatial recognition in the Y-maze was used as a hippocampal-dependent test [16] as part of a two-trial procedure, as previously described [17]–[20]. The apparatus was a Y-shaped maze made of gray plastic. Each arm was 34 cm long, 8 cm wide and 14 cm high. Extramaze visual cues were placed in the testing room and kept constant during the test. Because the three arms of the maze were identical, discrimination of novelty versus familiarity was only based on the different aspects of the environment that the mouse can perceive from each of them. The floor of the maze was covered with dirty sawdust from the home cages of several animals, and was mixed between sessions in order to eliminate olfactory cues. In the first trial, one arm of the Y-maze was closed with a guillotine door and mice (n = 9/experimental group) were allowed to visit two arms of the Y-maze for 5 min. After a 30 min inter-trial interval (ITI), the mice were again placed in the start arm for the trial 2 and allowed free access to all three arms for 5 min. Start and closed arms were randomly assigned to each mouse. Arm entries were defined as all four paws entering the arm. Preference for novelty was also measured using a 2-min ITI between acquisition and retrieval. This 2 min ITI was employed to control for potential motivational disturbances and verify that all groups made more visits to the novel arm when mnemonic demand was minimal [17]. The different ITI tests were separated by a 7-day interval. Analyses were based on the time spent exploring the novel and the familiar arms during the first 3 minutes of the trial 2 [21].

#### Object recognition

The visual object recognition test was used as a hippocampal-independent task [22]–[25]. Mice (n = 9/experimental group) were acclimatized in a 40×40 cm square cage made of white coated plywood with 16 cm-high walls for 15 min per day during the week before training. The floor of the maze was covered with corn cob litter which was mixed between each trial in order to remove olfactory cues. During a 10-min training period, two identical plastic blocks with particular shapes and colors were presented. After a 30 min ITI, one of the familiar blocks was replaced by a novel object, with a different shape and color, to test for memory retention. During the 5 min of testing, exploration of an object was defined as pointing the nose to the object and/or touching the object with the nose. Longer ITI (1-hour and 24-hour) were also employed to determine whether all groups recognized the novel object when retention was longer (data not shown) [22]. Analyses were based on the time spent exploring the novel and the familiar objects and the object recognition score was calculated as the difference in time spent exploring the novel and the familiar objects.

### IL-1β real-time PCR

WT and P2X_7_R^−/−^ mice were sacrificed with a lethal dose of pentobarbital immediately after completion of behavioral testing [2-min ITI or 30-min ITI Y-maze (Y-maze group) or free exploratory behavior (control group)]. Control-group mice were allowed to freely explore a new cage for the same amount of time as their spatially-trained counterparts (i.e. twice during 5 min each with either a 2-min ITI or a 30-min ITI) in the experimental Y-maze room. This control condition allowed the assessment of activation that results from exploratory behavior, sensorimotor stimulation, stress, and other factors not related to the spatial task. Hence, difference between the two profiles, *i.e.* the activation induced in the Y-maze group and in the control group, may reflect specific learning-induced changes.

Mice (n = 6/experimental group) were transcardially perfused with Phosphate Buffer Saline (PBS) and the hippocampi and hypothalamus were quickly removed and frozen in dry ice. Two µg of total RNA obtained from each brain area were reverse-transcripted with Moloney Murine Leukemia Virus reverse transcriptase (Invitrogen, Cergy-Pontoise, France). Quantitative PCR was then performed using the Applied Biosystems Assay-on-Demand Gene Expression Products protocol, as previously described [12]. Briefly, cDNAs for IL-1β and housekeeping gene (β2-microglobulin) were amplified by PCR by using an oligonucleotide probe with a 5′ fluorescent reporter dye (6-FAM) and a 3′ quencher dye (NFQ). Fluorescence was determined using an AB 7500 Real-Time PCR system (Applied Biosystems, Foster city, CA) and final quantification was performed using the comparative threshold (Ct) method as previously described [12]. For each experimental sample, the difference between target and housekeeping gene Ct values (ΔCt) was used to normalize for differences in the amount of total nucleic acid added to each reaction and in the efficiency of the RT step. Values were then expressed as relative fold change to the mean ΔCt value obtained for the group of control mice (free exploratory behavior, see below) (calibrator ΔCt) by subtracting ΔCt for each experimental sample from the calibrator ΔCt ( = ΔΔCt). The amount of target gene (linear value) normalized to the housekeeping gene and relative to the calibrator was determined by 2^−ΔΔCt^ (relative fold change: RFC).

In order to assess regional specificity of IL-1β induction, we analyzed IL-1β mRNA in the hypothalamus immediately after the completion of the 30 min ITI spatial recognition task using the same protocol as described above.

### c-Fos immunohistochemistry and immunocounting

WT and P2X_7_R^−/−^ mice (n = 6/experimental group) were sacrificed with a lethal dose of pentobarbital 90 min after the acquisition session of the spatial recognition task (Y-maze group) or free exploratory behavior (control group). This delay was chosen based on previous data showing that 90 min corresponds to the peak of the expression of c-Fos protein in the hippocampus in mice submitted to spatial memory tests [26]. WT and P2X_7_R^−/−^ mice of the Y-maze groups were subjected to a 5-min test in the Y-maze with 30-min ITI. Control-group mice were allowed to freely explore a new cage for the same amount of time as their spatially trained counterparts were given in the experimental Y-maze room (i.e. two 5-min periods with a 30-min ITI).

c-Fos expression was measured by immunohistochemistry as previously described [27]. After intracardiac perfusion with PBS (pH 7.4) followed by 4% paraformaldehyde, the brains were removed, post-fixed in the same fixative for 4 h at 4°C, cryoprotected in 30% sucrose for 24 h, and then quickly frozen in liquid nitrogen and stored at −80°C before sectioning. Coronal cryostat sections of 30 µm width through the hippocampus (bregma: from −0.9 mm to −3.1 mm) were collected free-floating. Briefly, rabbit polyclonal antiserum raised against c-Fos (Santa Cruz Biotechnology, Santa Cruz, CA) was diluted 1∶1000 in Tris Buffer Saline (TBS) containing 0.3% Triton X-100, 2% donkey serum and 1% bovine serum albumin (BSA), and sections were incubated overnight at room temperature (RT) before being incubated for 2 h with biotinylated donkey anti-rabbit antibody (1∶1000; Amersham Parmacia Biotech Europe, Freiburg, Germany), for 2 h with avidin-biotin peroxidase complex (1∶1000; Vectastain ABC kit, Vector laboratories, Burlingame, CA), and finally revealed with diaminobenzidine via the nickel-enhanced glucose oxidase method [28]. Sections were then slide-mounted onto gelatin-coated slides. The procedure also included negative controls with omission of the primary antibody, which did not show any immunoreaction.

Brain sections were examined under a light microscope with a 10× lens (Nikon Eclipse E 400) and the images were captured using a high-resolution digital Nikon DXM 1200 camera (Nikon Corporation, Champigny-sur-Marne, France). Camera aperture, magnification, light power and exposure time were fixed for all images. ACT-1-generated images (Nikon Corporation, Champigny-sur-Marne, France) were stored. Image-editing software (Adobe Photoshop, Adobe Systems, San Jose, CA) was used to adjust image size, brightness, and contrast. Quantification of c-Fos immunoreactive cells was performed with the aid of Scion Image public domain NIH-imaging software (Frederick, MD). The number of c-Fos-positive cells was quantified in the dentate gyrus (DG) and CA1 of the hippocampus from consecutive sections (n = 5–6 sections per mice; n = 6 mice per group) across both hemispheres, and these counts were averaged to produce a mean. Dorsal and ventral regions of the hippocampus were analyzed, but only the data of the dorsal region were presented as there were no significant differences in the ventral part. The number of c-Fos-positive cells was also quantified in the arcuate nucleus of the hypothalamus to control for specific hippocampal activation.

### Statistical analysis

All data are expressed as means±SEM. A two-way analysis of variance (ANOVA) with genotype (WT vs. P2X_7_R^−/−^) as between factor and arm or object (novel vs. familiar) as within factor were performed for spatial and object recognition analysis. Specific comparisons between novel and familiar (arms or objects) or between WT and P2X_7_R^−/−^ were assessed by paired and unpaired Student t-tests respectively. IL-1β mRNA and c-Fos quantification were analyzed by two-way ANOVA with genotype (WT vs. P2X_7_R^−/−^) and testing procedure (Control vs. Y-maze training) as between factors. When a significant interaction was reported, ANOVAs were followed by Fisher LSD post-hoc comparisons. Relations between recognition score (calculated as the difference between the investigation time for the novel and the familiar stimulus) and c-Fos levels were evaluated using Bravais-Pearson's correlation tests. The level of statistical significance was set at p<0.05.

## Results

### 1- Spatial memory performance is impaired in P2X_7_R^−/−^ mice

**Figure 1:**
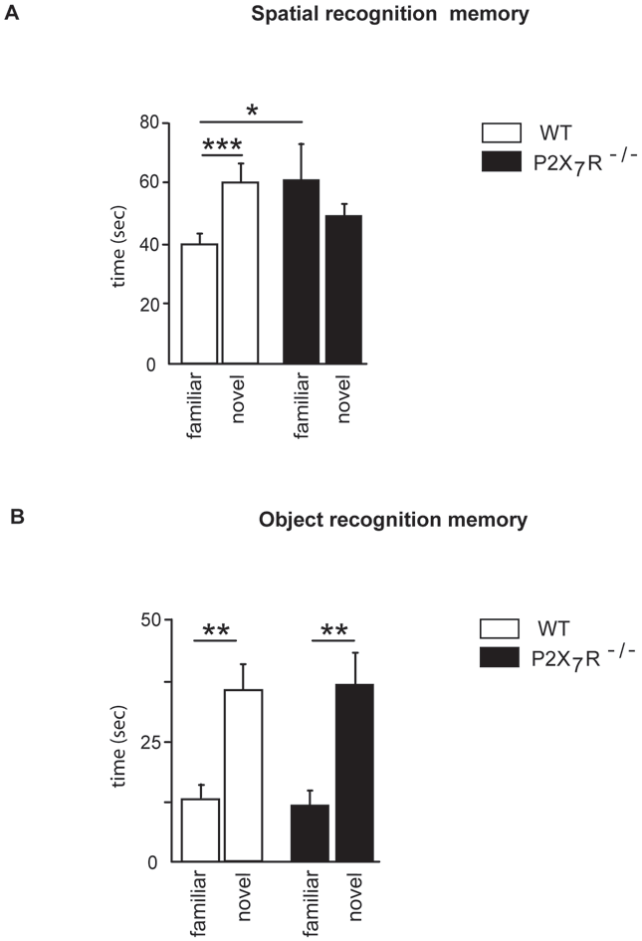
Recognition memory performance after 30 minutes of retention in P2X_7_R^−/−^ mice. (A) Time spent (sec) in the novel or the familiar arm after a 30-min ITI. (B) Time spent (sec) exploring the novel or the familiar object after a 30-min ITI. Spatial recognition memory, but not object recognition memory, was impaired in P2X_7_R^−/−^ mice after a 30-min ITI. ***p<0.001, **p<0.01,*p<0.05.

We first measured the spatial memory performances of WT and P2X_7_R^−/−^ mice using a spatial recognition test designed as a hippocampus-dependent task [16]. After 30 minutes of retention, the spatial memory performance was different across the genotype (Fig. 1A). A two-way ANOVA revealed no effect of genotype (F_(1,16)_ = 0.46, n.s.) or arm (F_(1,16)_ = 0.21, n.s.), but there was a significant interaction between genotype and arm (F_(1,16)_ = 4.5, p<0.05). Post-hoc analysis revealed that WT group spent less time in the familiar arm than P2X_7_R^−/−^ group (Fisher LSD, p<0.05). Further analysis indicated that WT mice significantly distinguished between the novel and the familiar arm (paired-t test, t(8) = 5.7, p<0.001), unlike the P2X_7_R^−/−^ group which exhibit a random exploration of the two arms (p = 0.42). No differences for the time spent in the starting arm between WT and P2X_7_R^−/−^ mice were measured (p = 0.176, not shown). In order to evaluate whether the impairment revealed in P2X_7_R^−/−^ mice was a memory deficit or a performance deficit related to motivational alteration, animals were tested in the spatial recognition test with a short ITI (2-min). When the mnemonic demand was minimal, all mice exhibited a clear preferential exploration of the novelty (arm effect, F_(1,16)_ = 17.2, p<0.001, no effect of genotype, F_(1,16)_ = 0.15, n.s., or arm x genotype, F_(1,16)_ = 1.64, n.s.; Table 1).

**Table 1:** Preference for novelty was measured using a 2-min ITI in WT and P2X7R^−/−^ mice.

	Preference for Novelty (ITI = 2 min)	
	Novel arm *(sec.)*	Familiar arm (sec.)***
**WT**	**84.28±8.84**	**36.82±6.60**
**P2X_7_R^−/−^**	**74.81±5.34**	**49.64±7.90**

Both strain exhibited a clear preferential exploration of the novelty (Arm effect, ***p<0.001).

We then measured performances of WT and P2X_7_R^−/−^ mice in an object recognition task designed as a hippocampus-independent task [22]. As shown on Fig. 1B, after 30 minutes of retention, all animals showed greater exploration of the new object (object effect, F_(1,16)_ = 41.6, p<0.0001, no effect of genotype, F_(1,16)_ = 0.01, n.s., or object x genotype, F_(1,16)_ = 0,09, n.s.), indicating that they readily recognized the familiar object (paired-t test, WT, t(8) = 4.52, p<0.01; P2X_7_R^−/−^, t(8) = 4.52, p<0.01). To evaluate whether longer ITI could reveal an impairment of object recognition in P2X_7_R^−/−^ mice, animals were tested with a 1-h or a 24-h ITI. As was the case with the 30-min ITI, all animals preferred the novel object over the familiar object with a 1-h ITI (object effect, F_(1,16)_ = 8.2, p = 0.01), whereas there was no discrimination of the novel object with a 24-h ITI (F_(1,16)_<1 for each effect; data not shown).

### 2- IL-1β mRNA expression after the spatial memory task is blocked in the hippocampus of P2X_7_R^−/−^ mice

The impaired behavioral performances of P2X_7_R^−/−^ mice in the spatial recognition task prompted us to investigate whether hippocampal IL-1β synthesis was altered in these mice. For this purpose, we measured IL-1β mRNA expression by real-time PCR in the hippocampus of WT and P2X_7_R^−/−^ mice immediately after the completion of two testing procedures: free exploratory behavior (control) or spatial recognition task (Y-maze with 2-min ITI or 30-min ITI).

**Figure 2:**
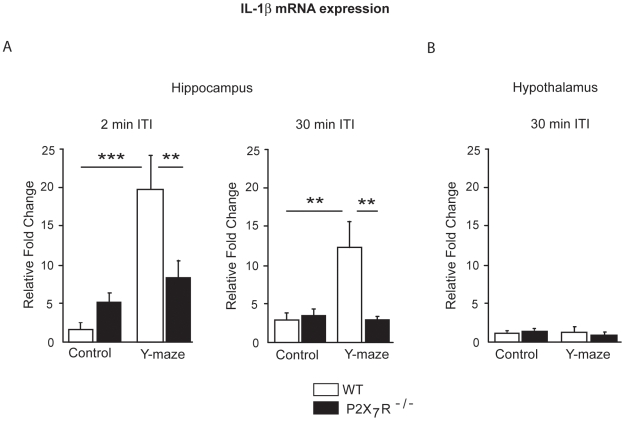
IL-1β mRNA expression after the exposure to the Y maze in P2X_7_R^−/−^ mice. IL-1β mRNA was measured by real-time PCR on WT and P2X_7_R^−/−^ mice sacrificed either after free exploration (control group) or completion of behavioral testing (Y-maze group). All data are expressed as mean relative fold change±SEM (see Materials and Methods for explanation). (A) IL-1β mRNA on hippocampus extracts from the 2-min ITI Y-maze group and their controls (left-hand panel) and the 30-min ITI Y-maze group and their controls (right-hand panel). (B) IL-1β mRNA on hypothalamus extracts from the 30-min ITI Y-maze group and their controls. Y maze exposure significantly increased the IL-1β mRNA expression in the hippocampus of WT mice but not of P2X_7_R^−/−^ mice. Whatever the group considered (WT or P2X_7_R^−/−^), no induction of IL-1β mRNA expression was detected in hypothalamus after the Y maze experience. ***p<0.001, **p<0.01.

Whatever the ITI, the Y maze experience increased IL-1β expression in the hippocampus (testing procedure effect, ITI 2-min, F_(1,20)_ = 17.7; p<0.001; ITI 30-min, F_(1,20)_ = 5.7; p<0.05). However, as shown on figure 2A, the IL-1β mRNA expression in the hippocampus was differentially affected by the Y maze exposure, depending of the group (genotype x testing procedure, 2-min ITI, F_(1,20)_ = 8.6; p<0.01; 30-min ITI, F_(1,20)_ = 4.4; p<0.05). *Post-hoc* analysis revealed that the effect of the exposure to the Y maze was exclusively observed in WT mice (control vs. Y maze, 2-min ITI, p<0.0001; 30-min ITI, p<0.01), but not in P2X_7_R^−/−^ mice (control vs. Y maze, 2-min ITI, p = 0.37; 30-min ITI, p = 0.84). Moreover, although there was no difference between WT and P2X_7_R^−/−^ in the control condition (2-min ITI, p = 0.33; 30-min ITI, p = 0.90), IL-1β mRNA expression was higher in WT mice than in P2X_7_R^−/−^ mice in the Y-maze condition (2-min ITI, p<0.01; 30-min ITI, p<0.01).

Next, we analyzed the impact of Y maze exposure on IL-1B mRNA expression in a brain area not involved in spatial memory (Fig. 2B). In order to assess the regional specificity of IL-1β induction, we analyzed IL-1β mRNA in the hypothalamus of the mice immediately after the completion of spatial recognition (Y-maze 30 min ITI) or free exploratory behavior (control). As expected, the Y maze exposure did not induce IL-1β mRNA expression in the hypothalamus either in WT or P2X_7_R^−/−^ mice (testing procedure effect, F_(1,20)_ = 0.98; n.s.; genotype, F_(1,20)_ = 0.83; p<0.01; genotype x testing procedure, F_(1,20)_ = 2.18; n.s.).

### 3- Spatial memory deficits in P2X_7_R^−/−^ mice are associated with blocked c-Fos activation in the hippocampus

**Figure 3:**
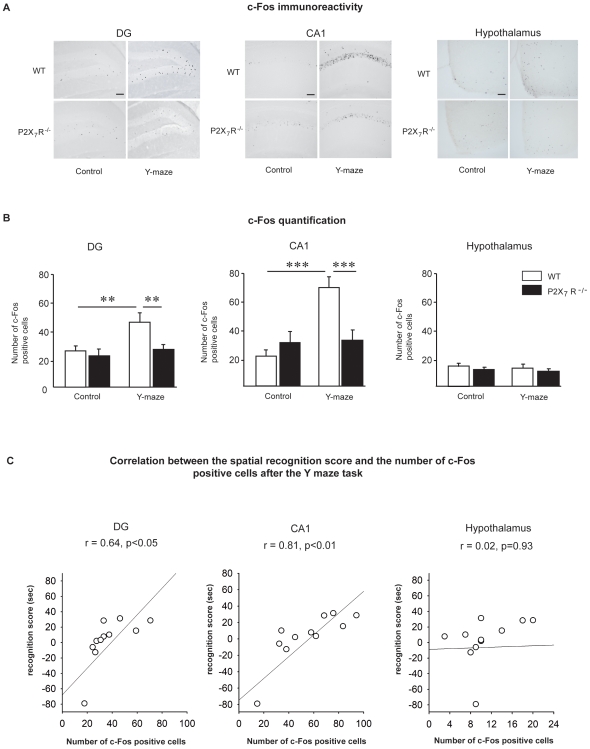
c-Fos expression after the exposure to the Y maze in P2X_7_R^−/−^ mice. c-Fos immunohistochemistry analysis was performed on the hippocampus and the hypothalamus of WT and P2X_7_R^−/−^ mice sacrificed 90 min after either the spatial recognition acquisition session (Y-maze group) or free exploration (control group). (A) Representative images of c-Fos immunochemistry in the DG (left-hand panel) and CA1 (middle panel) regions of the hippocampus and in the hypothalamus (right-hand panel). Scale bar = 100 µm. (B) Quantification of c-Fos-positive cells was performed in the DG (left-hand panel) and CA1 (middle panel) regions of the hippocampus and in the hypothalamus (right-hand panel). The spatial recognition in the Y maze task significantly up-regulated c-Fos expression in the DG and CA1 of WT mice but not of P2X7R^−/−^ mice. No increase of c-Fos expression was detected in hypothalamus after the Y maze experience. ***p<0.001; **p<0.01. (C) Correlation between the spatial recognition score (sec) and the number of c-Fos-positive cells induced by the Y maze task in the DG (left-hand), in the CA1 (middle panel) and in the hypothalamus (right-hand panel). Pearson's correlation coefficients (r) and corresponding significance (p) are displayed within each correlation windows. The spatial recognition performance in the Y maze after a 30-min ITI was positively correlated to the number of c-Fos positive cells in the DG and the CA1 of the hippocampus, but not with the number of c-Fos positive cells in the hypothalamus.

It has previously been shown that c-Fos immunoreactivity is a cellular activity marker that provides information on brain regions involved in learning and memory processes [29]. Because spatial memory performance and task-induced IL-1β mRNA expression in the hippocampus were impaired in our P2X_7_R^−/−^ mice, we sought to investigate whether cellular activity in the hippocampus had been affected. We therefore measured by immunohistochemistry, c-Fos expression in the hippocampus (DG, CA1) of WT and P2X_7_R^−/−^ submitted (Y-maze 30 min ITI) or not (control, i.e. free exploratory behavior) to a spatial recognition task (Figure 3A, B, left-hand panel). Two-way ANOVA revealed a significant effect of genotype (DG, F_(1,20)_ = 5.4; p<0.05; CA1, F_(1,20)_ = 4.5; p<0.05) and testing procedure (DG, F_(1,20)_ = 8.5; p<0.01; CA1, F_(1,20)_ = 14.9; p<0.001). However, the impact of Y maze exposure differed across the two groups of animals, as suggested by the trend towards genotype x testing procedure interaction in DG (F_(1,20)_ = 3.6; p = 0.07) and the significant interaction in CA1 (F_(1,20)_ = 13.4; p<0.001). Subsequent post-hoc analysis revealed that the spatial recognition task significantly up-regulated c-Fos expression in the DG and CA1 of WT (DG, p<0.01; CA1, p<0.001) mice but not P2X_7_R^−/−^ mice (DG, p = 0.47; CA1, p<0.89). Moreover, although there was no difference between WT and P2X_7_R^−/−^ in the control condition, c-Fos expression was higher in WT mice than in P2X_7_R^−/−^ mice in the Y-maze condition (DG, p<0.01; CA1, p<0.001). Assessment of c-Fos expression in the hypothalamus (Fig. 3B, right-hand panel) after the completion of spatial recognition did not show any effect of testing procedure either in WT or P2X_7_R^−/−^ mice (testing procedure effect, F_(1,20)_ = 0.96, n.s.; genotype x testing procedure effect F_(1,20)_ = 0.008, n.s.).

We then examined the relationship between the brain c-Fos expression after the Y maze and the spatial memory performance in this task. As demonstrated in the Figure 3C, c-Fos expression in the hippocampus was positively correlated to the spatial recognition score (DG, r = 0.64, p<0.05; CA1, r = 0.81, p<0.01). In contrast, no significant correlation was reported between the c-Fos expression in the hypothalamus and the memory performance (Figure 3C, right-hand panel, r = 0.02, p = 0.93).

## Discussion

We show here that compared to WT mice, P2X_7_R^−/−^ mice exhibit spatial memory deficits in the Y maze, a hippocampal dependent task, whereas their memory performance was intact in an object recognition paradigm. We further report that, contrary to the WT animals, P2X_7_R^−/−^ mice had no increase of hippocampal IL-1β mRNA and c-Fos expression after the Y maze exposure. Taken together, our results clearly support a crucial role for P2X_7_R-mediated IL-1β expression in memory processes involving the hippocampus.

Our study employed spontaneous recognition tasks (spatial and object), which exploit the natural tendency of rodents to explore novel place or stimuli more than familiar ones and have been widely validated as measure of recognition memory (reviewed in [16], [25]). These paradigms do not require the learning of a rule, and factors that may particularly influence performance in mice such as emotional states generated by commonly used procedures (food deprivation, water avoidance or electric foot-shock) are minimized, making it useful for studying memory in rodents [30]. Spontaneous recognition is based on novelty preference, however, when a delay of several minutes (e.g. 30 min), the decrease of the exploration of the familiar arm (or object) during the trial 2 reflects the memory of the previous exploration during the first trial [17], [19], [20], [30]–[33]. Following a 30-min ITI, WT mice showed significant spatial recognition, while P2X_7_R^−/−^ mice did not. Furthermore, P2X_7_R^−/−^ mice display intact performance in an object recognition task with the same 30-min ITI, suggesting that memory is not affected in a non-spatial task. Even if we cannot rule out that with longer ITI (such as 1h30 or 2h), the ability of mice to recognize object would decrease faster in P2X_7_R^−/^ mice than in WT mice, the differential impairment between spatial and object recognition tasks for a similar delay is particularly interesting regarding the considerable literature showing that spatial memory and object memory do not involved similar brain areas [22]–[25], [34]. Hippocampus is clearly involved for spatial memory; in contrast, visual/object recognition requires intact perirhinal area, but hippocampus seems less crucial [35]. Taken together, our results suggest that the lack of P2X_7_R specifically affects hippocampal functioning as well as the downstream events stemming from it that are specifically involved in spatial recognition. This possibility is well supported by the high expression of P2X_7_R both by presynaptic terminals of mossy fibers targeting CA3 pyramidal neurons [36] and by excitatory synaptic terminals in the CA1 and dentate gyrus regions of the hippocampus [37].

An important new finding of our study is the demonstration of the reduced expression of learning-induced IL-1β mRNA in the hippocampus of P2X_7_R^−/−^ mice. Interestingly, IL-1β was induced in the hippocampus but not in the hypothalamus, suggesting that IL-1β induction is specifically linked to spatial memory performance. This observation is consistent with previous reports showing that IL-1β gene expression is induced in the hippocampus by LTP in freely-moving rats [6], [38] and by two other hippocampal-dependent memory tasks, i.e. passive avoidance and contextual fear conditioning [5], [39]. Since, spontaneous recognition memory tasks do not allow a subtle assessment of memory processes (learning, flexibility, reference memory etc.), it would be interesting to evaluate whether the impaired spatial recognition memory in P2X_7_R^−/−^ mice can be generalized to other hippocampal-dependent tasks. In this context, it has previously been shown that IL-1 receptor activation is necessary for memory tasks such as passive avoidance, contextual fear conditioning or the Morris water maze [4]–[7].

IL-1β mRNA expression was induced in the hippocampus of WT but not P2X_7_R^−/−^ mice submitted to the spatial recognition task, independently of the ITI. These results suggest that P2X_7_R^−/−^ is a prerequisite for IL-1β mRNA expression, as previously demonstrated after LPS treatment [12]. In the hippocampus, P2X_7_R stimulation may either directly or indirectly activate neurons and elicit changes in their excitability [36], [37], [40]. Since an intact performance was reported in P2X_7_R^−/−^ mice after the 2-min ITI, despite a lack of IL-1β after the Y maze, we hypothesized that the induction of IL-1β in the hippocampus was an important factor underlying the spatial memory processes (e.g. retention of the spatial information), but not the spatial novelty preference. We did not identify the cell type that synthesizes and releases IL-1β in the hippocampus after the spatial task. Both neurons and microglia have been reported to express IL-1β in the non-pathological brain. There is a large body of evidence demonstrating the presence of P2X_7_R on microglia [8], [13], [41], [42]. The activation of these receptors, via downstream inflammatory stimuli such as LPS or direct activation by ATP, triggers microglial release of IL-1β, as we and others have previously demonstrated *in vitro* [8], [12], [13], [15], [41].

Similarly to IL-1β mRNA expression, c-Fos expression was induced in the hippocampus of WT but not P2X_7_R^−/−^ mice submitted to the spatial recognition task. c-Fos is an immediate early gene rapidly induced by a variety of stimuli, including stimuli involved in regulating synaptic plasticity [43]. This suggests that the increased neuronal activity in hippocampus observed after the exposure to the spatial task is linked to the hippocampal activation of P2X_7_R. Moreover, in our study, we found that c-Fos activation in the DG and CA1 of the hippocampus is positively correlated to spatial recognition performance. Indeed, mice exhibiting the best recognition scores also show the highest hippocampal c-Fos expression after the spatial task. In contrast, c-Fos levels in the hypothalamus were not linked to the spatial recognition score. The hypothalamus showed neither c-Fos expression nor IL-1β mRNA induction after the spatial task. This last observation further reinforces the link between P2X_7_R, IL-1β and c-Fos activation in the hippocampus in response to spatial memory performance.

IL-1β is synthesized in the cell cytoplasm as a leaderless procytokine (pro-IL-1β) which tends to accumulate in the absence of a maturation-promoting second stimulus. IL-1β-converting enzyme (ICE) is the key protease responsible for the intracellular processing of pro-IL-1β into mature IL-1β. Numerous *in vitro* and *in vivo* experiments using P2X7R^−/−^ mice have conclusively linked the stimulation of P2X_7_R by extracellular ATP to the activation of ICE and the downstream production of mature IL-1β [10], [14], [44]–[46]. Therefore, there is little doubt that a lack of P2X_7_R necessarily impacts IL-1β production, resulting in severe reduction if not a complete loss. Very importantly, IL-1β induced its own synthesis in the brain, and this process is dependent on P2X_7_R [12]. It is therefore possible that an initial small increase in IL-1β secretion through the recruitment of P2X_7_R could up-regulate the IL-1β mRNA induction in the hippocampus linked to a spatial recognition memory task. This idea is supported by the fact that contextual fear conditioning induces IL-1β mRNA expression in the hippocampus of WT mice but not of IL-1 receptor knockout mice [5]. The impaired memory processes in P2X_7_R^−/−^ mice could thus be linked to hippocampus-deficient IL-1β synthesis. Therefore, our results are in accordance with recent studies demonstrating that physiological levels of hippocampal IL-1β are required for short-term spatial memory performance, whereas deficient hippocampal IL-1β expression (as here) results in impaired memory.

The mechanisms underlying the role of P2X_7_R in the synthesis of IL-1β mRNA in relation to memory processes remain an enigma. P2X_7_R has been associated to IL-1β precursor processing, through the rapid activation of a specific caspase-1-dependent multiprotein complex [47]. The ATP-induced activation of P2X_7_R decreases cytoplasmic potassium which in turn induces the maturation and the release of mature IL-1β by monocytes [48], [49], microglia [12] and Schwann cells [48], [49]. In addition ATP by acting on presynaptic P2X_7_R, modulates hippocampal synaptic transmission [36], [37]. In the present work, it could be hypothesized that the Y maze exposure stimulates the ATP release by neighboring glial cells *via* an increase of the glutamate release [50]. This could in turn affect presynaptic P2X_7_R. Alternatively, or additionally, the memory task may modulate the neuronal release of ATP, which could then act on nearby P2X_7_R since ATP is co-stored and co-released with classical neurotransmitters from brain neurons [37], [51] in an exocytotic manner [52] and acts as a fast neurotransmitter within the brain [53]–[55]. However, up to now, the sole direct demonstration of P2X_7_R modulation of hippocampal synaptic transmission reported a depression [36].

In conclusion, our study demonstrated that P2X_7_R is involved in spatial memory processes and in the associated neuronal activity and IL-1β mRNA synthesis in the hippocampus. These results therefore corroborate clinical observations showing that the impairment of P2X_7_R function, due to mutation or polymorphism, leads to both immunological [56], [57] and brain dysfunction [58]–[60], putatively due to the lack of IL-1β synthesis and release.
